# Mechanisms of change in brief treatments for borderline personality disorder: a protocol of a randomized controlled trial

**DOI:** 10.1186/s13063-020-4229-z

**Published:** 2020-04-16

**Authors:** Ueli Kramer, Loris Grandjean, Hélène Beuchat, Stéphane Kolly, Philippe Conus, Yves de Roten, Bogdan Draganski, Jean-Nicolas Despland

**Affiliations:** 1 Institute of Psychotherapy and General Psychiatry Service, Department of Psychiatry, Lausanne University Clinic and University of Lausanne and Lausanne University Hospital, Place Chauderon 18, CH-1003 Lausanne, Switzerland; 2 General Psychiatry Service, Department of Psychiatry, Lausanne University Clinic and University of Lausanne, Lausanne, Switzerland; 3 Department of Psychology, University of Windsor, Windsor, Canada; 4 Department of Clinical Neurosciences, Lausanne University Clinic and University of Lausanne, Lausanne, Switzerland

**Keywords:** Psychiatric treatment, Randomized controlled trial, Mechanisms of change, Borderline personality disorder, Process, Emotional processing, Theory of mind processing, fMRI

## Abstract

**Background:**

Borderline personality disorder (BPD) is one of the most frequent, most debilitating and lethal mental conditions and is associated with a serious burden of disease. Treatment for patients with BPD involves structured psychotherapy, and may involve brief psychiatric treatment as first-line intervention. No controlled study has assessed the effectiveness of such brief intervention. Whereas most psychotherapy studies in patients with BPD focus on the effectiveness of the intervention, we still lack an understanding of how and why these effects are produced from a patient process perspective. It is therefore of utmost importance to study the treatment-underlying *mechanisms of change*. The present study plans to apply novel measurement methods for assessing change in two central psychobiological processes in BPD: emotion and socio-cognitive processing. The study uses theory-driven and ecologically valid experimental tasks, which take the patient’s individual experience as the anchor, by integrating methodology from psychotherapy process and neurofunctional imagery research.

**Methods:**

The aim of this two-arm, randomized controlled study is to test the effects (i.e., symptom reduction) and the underlying mechanisms of change associated with a brief psychiatric treatment (10 sessions over 4 months), compared with treatment as usual. Participants (*N* = 80 patients with BPD) undergo assessments at four points (intake, 2 months, discharge, and 12-month follow up). In addition to symptom measures, individuals undergo a 2-step assessment for the potential mechanisms of change (i.e., emotion and socio-cognitive processing): (1) behavioral and (2) (for a sub-sample) neurofunctional. We hypothesize that change in the mechanisms explains the treatment effects.

**Discussion:**

This study uses an easy-to-implement treatment of BPD, and a sophisticated assessment procedure to demonstrate the critical role of psychobiological change in emotion and socio-cognitive processing in brief treatments. It will help increase the effectiveness of brief treatment for BPD and help diminish the societal burden of disease related to BPD, in these early stages of treatment.

**Trial registration {2}:**

ClinicalTrials.gov: NCT03717818. Registered on 24 October 2018). Protocol version {3} number 2 from 9 February 2018.

## Administrative information

**Table:** 

Title (1)	Mechanisms of change in brief treatments for borderline personality disorder: a protocol of a randomized controlled trial
Trial registration {2a and 2b}	Clinical Trials NCT03717818
Protocol version (3)	Number 2 from February 9^th^, 2018
Funding {4}	This trial is supported by the Swiss National Science Foundation (SNSF100014_179457/1, to Dr. Kramer).
Author details {5a}	Ueli Kramer, Loris Grandjean, Hélène Beuchat, Stéphane Kolly, Philippe Conus, Yves de Roten, Bogdan Draganski, Jean-Nicolas Despland
Name and contact information {5b}	Swiss National Science Foundation (SNSF), Wildhainweg 5, Postfach 8232, CH-3001 Bern, Switzerland. Phone: +41 31 308 22 22. E-mail: div1@snf.ch
Role of sponsor {5c}	The Swiss National Science Foundation (SNSF) is funding this project,but does not have any influence on any level of content or procedure of this project. It is the role of the SNSF to ensure that proper arrangements are in place to initiate, manage and report on a study. SNSF also ensures that appropriate indemnity is in place before research begins.

## Background and rationale {6a}

Borderline personality disorder (BPD) is one of the most prevalent mental disorders with a prevalence of 2–3% in the general population. Direct societal costs are related to frequent use of emergency services, intense use of inpatient and outpatient treatments, indirect societal costs stemming from prolonged sick leave, abusive consumption of street-drugs, intra-familiar abuse and neglect and in some cases legal costs [1, 2].

Psychological treatments are considered first line for problems related to personality disorders [3–6]. Although there are several theoretical accounts on how these effects are produced, there is a paucity of systematic empirical research focusing on mechanisms of change in treatments of personality disorders (PDs) [7–13], aiming at *empirically explaining* how treatments work and, ultimately, *increasing* the treatment’s effectiveness. Such an empirical understanding would be particularly useful at the beginning of treatment: a better knowledge of the determinants of initial symptom alleviation in psychological treatment would allow us to deliver even more potent treatments for these patients from the very first session on, and to prevent some of the long-term consequences of the disorder.

The objective of the present study is to explain early symptom change in patients with borderline personality disorder undergoing a brief psychiatric treatment that is consistent with the international and national treatment guidelines [3, 14]. We assume that symptom change is the result of a complex interplay between changing central process characteristics of the patient - assessed from an integrated neuro-behavioral perspective - and the moment-by-moment responsive adjustment to them by the therapist. The present trial examines two patient-related mechanisms of change in treatments of BPD: (1) emotion processing and (2) change in socio-cognitive processing. Aimed at discovering the underlying “laws of change” in patients undergoing brief treatments for BPD, such research may help increase the effectiveness of any bona fide therapy approach from the very first session on and thus may help decrease direct and indirect societal costs related to BPD.

In order to achieve this, we aim to study a specific brief guideline-based intervention for BPD. In addition to the structured psychotherapy models validated for the treatment of BPD (e.g., dialectical-behavior therapy (DBT), mentalization-based therapy (MBT), transference-focused psychotherapy), the past 10 years have witnessed the emergence of psychiatric approaches to the treatment of BPD [15, 16]. In a randomized controlled trial, McMain and colleagues [17, 18] have tested the efficacy of a 1-year-long dialectical-behavior treatment program compared to a general psychiatric management (GPM) program, also lasting 1 year [19, 20]. The results were somewhat unexpected, showing that both treatments performed equally well on all outcome indices and most process characteristics, including symptom relief up to 2 years after the end of treatment. Bateman and Fonagy (2009) used a similar type of psychiatric approach in which they found consistent results with regard to the comparison with MBT [21]. These emerging studies may suggest that for at least the first phase of treatment of patients with BPD, it might be sufficient to offer a psychiatric intervention that is easily learnable; as such, we increase general access to mental health intervention for a large amount of patients with BPD [22, 23]. Such new healthcare models have been advocated within the literature on stepped care for BPD [24–26].

To date, no study has examined such a brief guideline-based psychiatric intervention, together with its explanatory mechanisms of change for outcome, compared to treatment as usual. This is the aim of the present research. It will determine the effectiveness of a brief psychiatric intervention and detail its psychobiological underpinnings of change.

### A methodological problem and a possible solution

Earlier studies focusing on mechanisms of change in treatments for BPD fall into two major categories, neglecting a possible methodological integration explaining psychobiological change. Some studies have involved psychotherapy process research, describing change on central patient variables in-session [27–29] favoring external clinical validity, but remaining sensitive to the session-internal influences by the patient-therapist interaction (i.e., therapist responsiveness) [30]. Other studies have involved biological assessments of change where the changing variable was observed on neurobiological activation to the exposed standardized stimuli [31, 32] - favoring internal validity, but neglecting the idiographic content so central to understanding change in psychotherapy.

In order to address these methodological problems, we suggest researchers should carefully integrate methods from psychotherapy process research with neurofunctional methods, by taking into account the *individual’s subjective experience* as the anchor - substantiated in the form of *individualized* stimuli in the experiment - in the assessment of the mechanisms [12, 33]. This is only meaningful when the design controls for a number of manipulation checks (see “Methods”). Such an integrated experimental design goes beyond the systematic assessment of the therapy process, as described by our earlier studies [28, 34, 35]; in addition, it becomes possible to relate aspects of the patient’s subjective experience to the neurobehavioral correlates of change (see also the discussion by Tashiro et al., 2006, and Kramer, 2019 [12, 36]).

### Mechanisms of change in treatments for borderline personality disorder

Here, we subsequently focus on two potential mechanisms of change that are among the most potent so far, and embed them within our integrative conception of mechanisms of change: (1) emotion processing and (2) socio-cognitive processing.

### Emotion processing: change in affect-meaning states related to self-criticism

Change in emotion processing is central for psychotherapy in patients with BPD [37]. Greenberg and colleague (2006) have differentiated between, among others, emotion regulation and emotion transformation [38]. With regard to emotion regulation over the course of psychotherapy, Neacsiu and colleagues (2010) showed that the acquisition of specific coping-skills strategies, aiming at more efficient emotion regulation, functioned as a mediator of change in DBT [39]. These findings are in line with the results of a pilot functional magnetic resonance imaging (fMRI) study: Schnell and Herpetz (2007) investigated cognitive reappraisal over the course of inpatient DBT in six patients with BPD [31]. The authors identified a decrease in activation over the course of therapy at the levels of the right anterior cingulate cortex (ACC), the temporal and posterior cingulated cortex and the left insula [31]. Using a previously validated procedure of cognitive reappraisal [40], Schmitt and colleagues (2016) partially confirmed these results in a sample of patients with BPD (*N* = 32 patients) undergoing DBT and identified enhanced neural connectivity between ventro-lateral pre-frontal cortex areas and the amygdala [32], consistent with the neuropathological model put forward by New and colleagues (2007) [41]. Comparable results were obtained in 11 patients with BPD undergoing DBT, compared with 11 healthy controls: decreased amygdalar reactivity was identified after DBT [42, 43]. Consistent results were identified for change in distraction as a strategy for regulation of emotion [44] and change in pain-mediated regulation of emotion [45]. Perez and colleagues (2015) identified consistent effects in favor of enhanced fronto-limbic connectivity after 1-year long transference-focused psychotherapy (TFP) in 10 patients with BPD [46]. Taken together, these results suggest improved emotion regulation capacities after therapy in patients with BPD, but more research integrated within a larger theoretical framework is needed.

In addition to the regulation perspective on emotion, we define *emotion transformation* as the change process by which emotions unfold and change over time from the least productive to the most productive, the latter being underpinned by increased meaning-making [47]. It was shown that this process relates to healthy functioning and a good outcome from psychotherapy [34], in particular the flexibility of emotional experiences [47]. The relevance of this dynamic conception of emotion processing to BPD was supported in terms of change in anger processing [48] and in terms of change in undifferentiated global distress [27]. It was shown to also be of relevance in narcissistic and histrionic PDs [49, 50].

This transformation conception may explain the resolution of harsh self-criticism, a central clinical feature of BPD [48, 51–53]. Whelton and Greenberg (2005) proposed a paradigm of studying emotion transformation related to self-criticism using the empty-chair dialogue. Patients criticized themselves in a structured assessment procedure using imaginative and emotion-eliciting enactment tasks. It appeared that the depressive persons presented more self-contemptuousness in their self-criticism, compared to controls and presented with higher levels of shame, sadness and emotional collapse, along with less pride [54]. This study was on 45 undergraduates presenting with or without anger problems, using the same paradigm [49]. What differentiated the two groups was the presence of self-contemptuousness, *t* (1, 43) = 1.91, *p* < .05) associated with the self-criticism, along with the absence of the existential need in the anger-prone individuals. This means that for anger-prone participants - who share this clinical feature to some extent with BPD - self-criticism is particularly harmful to the emotion transformation when associated with self-contemptuousness. We may therefore assume that decrease in self-contemptuousness - and possibly increase in its antidote, self-compassion - and increase in emotion flexibility are markers of productive change in treatment.

Emotion transformation related to self-criticism is underpinned by biological changes. Using standardized stimuli, Longe et al. (2010) showed in a female student sample (*N* = 17) a blood oxygen level dependent (BOLD) activation (intra-subject comparison to a neutral condition at the level *p* < .05 corrected) in the left pre-frontal cortex (PFC; Brodman area (BA) 45), in the lateral orbito-frontal cortex (OFC; BA 47), in the left dorsolateral PFC (BA 9) and in the inferior and middle temporal gyrus (BA 20 and 21, including the lingual gyrus, BA 19) [55]. The hyperactivity in pre-orbito-frontal and orbito-frontal regions associated with self-criticism in this study was interpreted as linked with the inhibitory behavior known to be associated with activation of the lateral PFC [56]. Brain activity in the striatum has been associated with self-punishing emotions of self-criticism [54], such as shame, anger about the self and self-contemptuousness [35]. In addition, some regions of the insula-basal ganglia networks are known to be associated with processing of disgust [57]. In an fMRI study using *individualized* self-critical stimuli (which were previously selected based on a large set of words), Doerig and colleagues (2013) showed bilateral insula activation, along with activations in left hippocampus and amygdalar formations, interpreted as regions recruited in emotion processing of self-critical stimuli [58]. More research is needed to understand change in self-contemptuousness and its neuronal substrates over the course of treatment for BPD, when an individualized measurement method is applied.

### Change in socio-cognitive processing: integrating “hot” interpersonal information

Change in the patient’s *socio-cognitive or mentalizing capacities* is discussed as a putative mechanism of change in the treatment of BPD [52, 59, 60]. Levy and colleagues (2006) examined change in three forms of psychotherapy for BPD -– TFP, DBT and supportive therapy - and found that only TFP was associated with an increase in mentalizing functions, along with development of more secure attachment patterns in some patients in this group [29]. Consistent results were presented by Fischer-Kern and colleagues [61] (see also de Meulemeester et al. [62]; Maillard et al., [63]). Other research has underlined the moderating factor of mentalizing capacities for outcome for different categories of PD [64, 65]. To our knowledge, no studies have shown mediation for changes in socio-cognitive processing in treatments for BPD.

One method to investigate the core interpersonal contents related with attachment figures (i.e., “hot” stimuli), again formulated from an *individualized* perspective, is the core conflictual relationship theme (CCRT [66]). A CCRT is a formulation composed by a patient’s wish (e.g., to be close, to be treated harshly), the anticipated response of the other/the object (e.g., to facilitate one’s independence, to be harsh) and the response of the self (e.g., to feel understood, to be frustrated). According to Luborsky (1998), the *pervasiveness* of a CCRT is the degree of generality of a theme across specific relationship episodes and specific interactions [66]. In patients with BPD, one may identify a central theme for each person, which is present in more than 60% of the specific relationship episodes [67, 68]. After treatment, it is expected that pervasiveness related to the core theme decreases. Luborsky (1998) demonstrated in 33 patients undergoing psychodynamic psychotherapy - although not patients with BPD - a pre-post decrease in pervasiveness over time (*F* (1, 32) = 7.4, *p* < .01), which was particularly strong for the category of the negative response of the self. This decrease correlated with symptom change at the end of treatment [66]. Therapy studies in patients with BPD are needed, to test the role of decrease in CCRT pervasiveness over time.

As such, an emerging field of research focuses on the explanatory roles of (1) the patient’s emotion processing and (2) the patient’s capacities of socio-cognitive processing, for treatment effects related to PDs. We think that the most promising assessments rely on integration between idiographic and nomothetic assessment procedures.

### Objectives {7}

(1) Outcome: a 10-session BPD-specific guideline-based treatment produces more reduction in specific borderline symptoms than non-specific treatment as usual (TAU). (2) Global change: a 10-session BPD-specific treatment presents pre-post change in socio-cognitive processing (SCP) and emotion processing (EP), which is not the case in the TAU. (3) Treatment response: SCP and EP change more in treatment responders, compared to non-responders (across conditions). (4) Mediation: the changes in these potential mechanisms of change will function as mediators of symptom decrease (between intake, discharge and follow up; the latter will be used to disentangle potential time confounds). Change is operationalized on behavioral and neurofunctional (by controlling for the corresponding behavioral/idiographic information) levels. For post-therapy EP, we expect greater emotional variability when dealing with individualized self-criticism, along with lesser self-contemptuousness, compared with pre-therapy. It is expected that post-therapy neuronal activations are lower in a EP network involving areas in the PFC, the striatum and the insula-basal ganglia [57, 58], when compared with pre-therapy. For post-therapy SCP, we expect lower CCRT pervasiveness, when compared with pre-therapy and we expect that post-therapy neurofunctional activations are lower in regions associated with the theory of mind (i.e., anterior cingulate, precuneus, inferior and middle frontal gyri and inferior parietal lobes) [69–71], compared with pre-therapy.

### Trial design {8}

The present study is planned as a randomized controlled treatment trial, involving a guideline-based psychiatric treatment (general psychiatric management (GPM) [20]) for BPD, over the course of a program comprising 4 months of treatment plus 12 months of follow up, in comparison with 4 months of TAU, by focusing on the underlying mechanisms of change. The outcome part is a superiority trial, while the mechanisms part focuses on controlled comparison of processes of change.

## Methods: participants, interventions, outcomes

### Study setting {9}

The trial takes place in the context of Lausanne University Hospital and the University of Lausanne, Switzerland.

### Ethical clearance

With respect to the submitted project on the Swiss Ethics platform, the competent Canton de Vaud Ethics Committee, has approved the study (2017–02167). The study is registered (NCT0317818). In keeping with the established and approved data management plan, only anonymous data will be kept in the file. All raw video data, where the patient may be identified, and fMRI data will be stored separately from patient identifiers and from the main dataset.

### Eligibility criteria {10}

Patients with BPD according to the *Diagnostic and statistical manual of mental disorders, fifth edition* [72] and who have sufficient mastery of French will be included. The rate of comorbidity is expected to be high. All patients who accept participation in the study by giving informed written consent will be included in the outcome and mechanisms parts of the study; a sub-group of patients who fulfil additional inclusion criteria (female, younger than 45 years, right-handed, on stable medication or no medication and absence of formal counter-indication on the security check) will be included in the fMRI part of the study.

Patients with neurocognitive disorder, psychosis and bipolar disorder I will be excluded from the trial. In order to ensure generalizability of the results to a wide variety of clinical settings, no other exclusion criteria will be applied.

### Who will take informed consent? {26a}

After the patient makes a request for treatment at the Department of Psychiatry, University of Lausanne, for problems related to BPD, the patient meets with a researcher who explains to him/her the study and informs him/her about the randomization and the assessment schedule. On the consent form, participants will be asked if they agree that their data be used should they choose to withdraw from the trial. Participants will also be asked for permission for the research team to share relevant data with people from the Universities taking part in the research or from regulatory authorities.

### Additional consent provisions for collection and use of participant data and biological specimen {26b}

This trial does not involve collecting biological specimens for storage {26b; 33}. The relevant consent form can be obtained from the corresponding author upon request {32}.

## Interventions

### Explanation for the choice of comparators {6b}

In order to test the hypotheses, a brief version of Good Psychiatric Management (GPM) is compared with a brief version of treatment of usual. This is in keeping with standard methodological recommendations in the study of effectiveness of psychotherapeutic interventions.

### Intervention description {11a}

Brief treatment encompasses the communication about diagnoses, problem areas, anamnesis, the work on treatment focus, objectives and motivation, the treatment of treatment-interfering problems and the elaboration of interpretations related to the core concept of interpersonal hypersensitivity, according to the principles of GPM for BPD [20, 73–75]. For the TAU, 10 therapists will intervene using non-specific crisis management as usual (minimal ethical assessment and contact with the patient according to the local directives, ensuring safety management).

### Criteria for discontinuing or modifying allocated interventions {11b}

Should a participant request discontinuation of the intervention, the study therapist will use this as criteria for discontinuing and modifying the allocated intervention.

### Strategies to improve adherence to interventions {11c}

Therapist adherence to the protocol will be self-assessed by the therapist using Gunderson’s (2016) questionnaire for adherence to GPM principles, to be filled in after each treatment.

### Relevant concomitant care permitted or prohibited during the trial {11d}

Relevant concomitant care was permitted during the trial, and was recorded.

### Provisions for post-trial care {30}

The data management plan (see below) outlines procedures in the case of adverse events in the context of the trial, which includes provision, if needed of post-trial care in the case of harm.

### Outcomes {12}

The main outcome measure is the Zanarini Rating Scale for Borderline Personality Disorder (ZAN-BPD) [76]. The ZAN-BPD is a continuous hetero-administered measure assessing the nine criteria outlined in DSM-5, on a continuous Likert-type scale ranging from 0 to 4. As such, it yields a total theoretical score of 36. A comprehensive validation study has shown its reliability, validity and sensitivity to change [76].

Emotion processing related to self-criticism will be assessed using the self-criticism task. This task involves two main steps: (1) conduct of a two-chair dialogue on self-criticism, an individualized and therefore particularly emotion-arousing procedure [35, 54, 77] and observer’s process rating of the patient’s emotions using the Classification of Affective Meaning States (CAMS) [78] (see also [34, 47]) with the aim of extracting 20 individualized self-critical words for each patient at each assessment point. Increase in emotion flexibility (i.e., more different emotion categories as a reaction to the self-criticism) over time, along with a decrease in self-contemptuousness associated with the self-criticism over time, are indicators of productive change; (2) 1 week later, a test of neural correlates of the processing related to the 20 individualized self-critical words (extracted from step 1), in comparison with a set of 20 negative emotional [79], 20 positive emotional [79]), 20 neutral words and 20 non-words (symbols; in total 100 words; presented in a random order). Self-reported emotional arousal (on a self-assessment manikin (SAM)) and self-esteem (on the State Self-Esteem Scale (SSES)), along with observer-reported fear/shame (on the CAMS) reaction, will be measured as manipulation checks for step 1; SAM assessment will be performed for each stimulus presented in the scanner. This task was empirically tested in a non-published pilot study: in 5 individuals, we showed higher subjective arousal levels for the individualized words than for the standardized negative words, along with comparable neurofunctional activations.

**Fig. 1:**
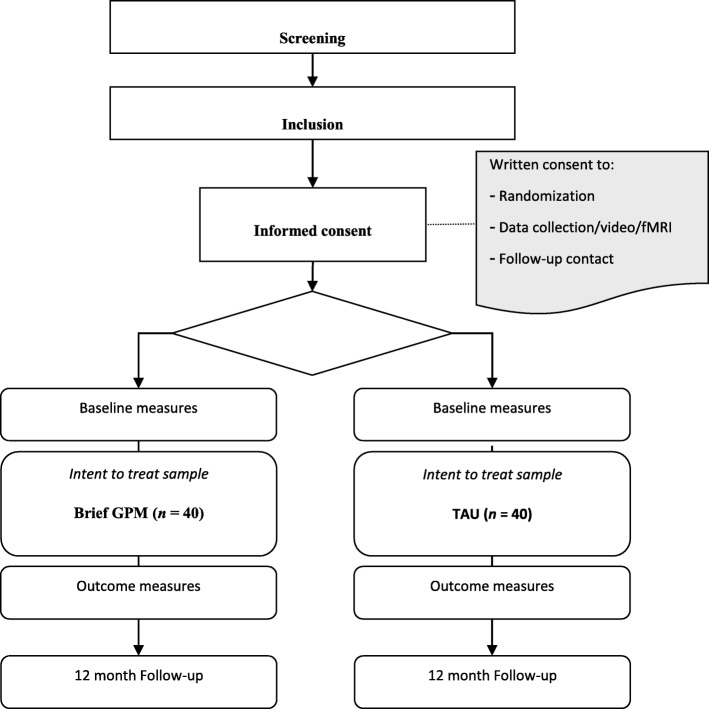
Consolidated standards of reporting trials (CONSORT) flowchart of the research procedure. fMRI, functional magnetic resonance imaging; GPM, Good Psychiatric Management; TAU, treatment as usual

Change in socio-cognitive processing of interpersonal patterns will be assessed using two independent tasks, one behavioral, and the other fMRI. The behavioral task involves the conduct of a structured interview using the Relationship Anecdote Paradigm [66] and based on this video-taped structured material, the observer’s process rating of the patient narrative using the CCRT [66–68] with the aim of comparing its pervasiveness pre-follow-up to post-follow-up (see Fig. 1). Decrease in CCRT pervasiveness is an indicator of productive change. The fMRI task involves the appreciation of humoristic stimuli measuring the patient’s theory of mind; this task has previously been validated for BPD [69] (for the validation of the stimuli see [80, 81]). It involves the processing and understanding of three sets of stimuli, presented in a pseudo-random order: (1) theory of mind (ToM): visual jokes requiring attribution of false mental states to the protagonists presented in the cartoons (30 stimuli); (2) visual puns (PUN): visual puns, i.e., cartoons that are based on visual similarities, not requiring attribution of false mental states (30 stimuli) and (3) a non-humorous control condition with incongruent visual information (30 stimuli, in total *N* = 90). Manipulation checks involve the assessment of the understanding of each joke. Decrease in activation of the ToM network over time is an indicator of productive change.

The remaining assessments concern secondary outcomes and include the Outcome Questionnaire-45 [82], which is a self-report questionnaire comprising 45 items aiming at assessing psychotherapeutic results, including a global score and three sub-scale scores: symptomatic level, interpersonal relationships and social role. It has been translated and validated in French [83]. The Borderline Symptom List (BSL-23) [84] is a self-report questionnaire assessing the BPD symptomatology using 23 items; it represents a short version of the more extensive Borderline Symptom List [85] for which excellent psychometric properties were reported. Similar results were found for the short version used in this study [84]. The French version has shown comparable validity coefficients [86]. The Inventory of Interpersonal Problems (IIP) [87] (French translation by Stigler) is a self-report questionnaire assessing interpersonal patterns on several dimensions, such as affirmation, affiliation, submission, intimacy, responsibility and control. In total, this questionnaire comprises 64 items*.* Spielberger State-Trait Anger Inventory (STAXI-2) [88] is a self-report questionnaire on trait and state anger, using 44 items. This was translated to French and adapted by Borteyrou, Bouchon-Schweitzer and Spielberger [89]. The Difficulties in Emotion Regulation Scale [DERS] [90] is a self-report questionnaire assessing the quality of emotion regulation using 36 items. The French translation and validation of this instrument yielded a satisfactory factor structure on a student sample [91]. As a measure of the therapeutic alliance, the Working Alliance Inventory (WAI) [92] (French validation [93]) will be given after each session; the therapeutic alliance will be introduced as moderator where appropriate. At intake, reliable psychiatric diagnoses (using the Structured Clinical Interview for DSM-5 (SCID-5-CV) and Structured Clinical Interview for Personality Disorders (DSM-5) (SCID-5-PD) developed by the American Psychiatric Association), childhood trauma (Childhood Trauma Questionnaire [94]; French version [95]), rejection sensitivity (Rejection Sensitivity Questionnaire-Adult [96]) and the level of intelligence (National Adult Reading Test (NART), French version [97]) will be assessed.

Manipulation checks will be introduced by using self-report questionnaires of arousal (SAM [98]), self-esteem (SSES [99]) and vividness (Vividness of Visual Imagery Questionnaire (VVIQ) [100, 101]).

### Participant timeline {13}

Assessments take place at intake, 2 months and discharge, plus at follow up after 12 months. At follow up, the participants will be contacted by the research team; see {18b}. Importantly, any participant who dropped out of treatment will still be contacted for research assessments and follow up.

### Sample size {14}

Based on power analysis (presumed power 0.819, for two concomitant mechanisms, *d* = 0.60; two-tailed alpha = .05; 30% dropout), we plan to recruit 80 patients with BPD for this study at the Department of Psychiatry, University of Lausanne, Switzerland. In total, 10 therapists (psychiatrists or psychologists) will participate in the study for the GPM treatment; they have had at least 6 h of specific training in psychiatric treatment for BPD [102], in addition to training in psychotherapy according to Federal regulations.

### Recruitment {15}

Participants will be recruited at the Department of Psychiatry, University of Lausanne, Switzerland, in a specialized clinic for the treatment of BPD. The current patient flow will guarantee recruitment in the protocol. Patients are paid CHF70 for full participation in an assessment point.

## Assignment of interventions: allocation

### Sequence generation {16a}

A system involving computer-generated random numbers (block randomization in blocks of 10) was used at the outset and the allocation numbers were put into sealed envelopes. The latter are opened by the researcher upon patient inclusion. Patients are not stratified a priori.

### Concealment mechanism {16b}

The sequence is concealed within sealed envelopes, which were prepared by a trial-independent researcher.

### Implementation {16c}

The allocation sequence is generated by the computer, the concealment by an independent researcher. Then the study researchers enroll the participants and assign participants to interventions, after the opening of the sealed envelope.

## Assignment of interventions: blinding

### Who will be blinded {17a}

The assessors (of outcome and mechanisms) and data analysts will be blind to the experimental condition, but the patients and the therapist will not. In order to control for bias, assessors will be polled at the end of the study with regard to each patient’s condition.

### Procedure for unblinding if needed {17b}

The competent Ethics Committee can request audits at any moment in time and the primary investigator and his team will follow its instructions. Audit may include to disclose, in a restricted fashion and only if deemed necessary, personal data related to participants to the Ethics Committee.

## Data collection and management

### Plans for assessment and collection of outcomes {18a}

All relevant information related to the reliability and validity of the outcome measures are provided under assessments {12}. All interviewers, assessors and raters are extensively trained in the relevant assessment procedures, and reliability is checked continuously. All data are collected in a secure online system, which assures privacy protection and data integrity, as described in the data management plan (see below).

### Plans to promote participant retention and complete follow up {18b}

At follow up, the participants will be contacted by the research team. Importantly, any participant who dropped out of treatment will still be invited for research assessments and follow up.

### Data management {19}

According to the accepted data management plan, data will be entered into RedCap on a secure space on the University server. This program allows full accountability of data management, and standard procedures are in place should problems arise. All details are found in the data management plan, which can be obtained from the primary investigator upon request.

### Confidentiality {27}

Data safety is guaranteed by the system’s security check and no formal data monitoring committee is requested. Participants’ personal information will be stored at a separate, locked, location at the Department of Psychiatry. No personal information will be revealed before, during or after the trial (except in the case of audit by the competent Ethics Committee; plans to give access to the protocol, participant level-data and statistical code are described in the data management plan; see {31c}).

### Plans for collection, laboratory evaluation and storage of biological specimens for genetic or molecular analysis in this trial/future use {33}

This trial does not involve collecting biological specimens for storage.

## Statistical methods

### Statistical methods for primary and secondary outcomes{20a}

For the behavioral assessments and all outcomes, we will conduct intent-to-treat and completer analyses for all variables (hypothesis 1: outcome, defined as residual gains at discharge). We will use multilevel modeling [103] where appropriate, for hypotheses 2 and 3 (global change and treatment response). For hypothesis 4 (mediation), we will conduct a mediation analysis for both potential mechanisms of change [104]. Raw and composite scores for outcome and all mechanisms of change indexes will be used, by controlling for the corresponding fMRI data from the same assessment point. Composite scores for EP involve combining pre-post change in contemptuousness with change in neuronal regions of interest for individualized self-criticism. For SCP, this involves combining pre-post change in CCRT pervasiveness with change in neuronal regions of interest for theory of mind.

We will control for therapist effects in the three-level hierarchical linear modeling (HLM) [105]. All indexes (i.e., behavioral and fMRI) in the patient groups (*N* = 80) at intake will be rigorously compared with the indexes found in the healthy control group (*N* = 20); we expect systematic between-group differences, in the context of a control analysis. Statistical treatment packages HLM7 and SPSS23 will be used for the analyses of the behavioral indexes.

For the fMRI assessments, we will use the methodology of blood-oxygen-level-dependent (BOLD) imaging followed by standard data processing and statistical analysis in the framework of Statistical Parametric Mapping software (SPM12). The fMRI data will be acquired on a Siemens Prisma 3 T (64-channel head coil using a 2D echo planar (EPI) sequence). The acquisition parameters will be as follows: 3 × 3 × 3 mm3: echo time (TE) =30 ms, slice repetition time (TR) = 66 ms, 30 slices, flip angle = 90°. The structural MRI data consist of T1-weighted MPRAGE images (TR = 2000 ms; inversion time (TI) = 920 ms; α = 9°; bandwidth (BW) = 250 Hz/pixel; readout in inferior-superior direction; field of view (FoV) = 256 × 232 mm; 176 slices) at 1 mm resolution. All data pre-processing will be performed using the SPM12 (Wellcome Trust Centre for Neuroimaging, shttp://www.fil.ion.ucl.ac.uk/spm/) running under Matlab 7.13 (The MathWorks, Inc., Natick, MA, USA). EPI images will be realigned to the subject’s average image across trials (corrected for spatial distortions using the SPM fieldmap tools). The parameters of registration to standardized Montreal Neurological Institute (MNI)-defined space will be calculated on the anatomical image and the default settings of the “unified segmentation” framework followed by the diffeomorphic registration algorithm DARTEL [106, 107]. The spatial registration parameters will be applied to the functional time-series co-registered to the corresponding individual’s anatomical scan. Prior to statistical analysis, we will apply spatial smoothing with a Gaussian kernel of 8 mm full-width-at-half-maximum. All statistical analyses will be performed using the default settings in SPM12. The statistical analysis at subject-specific level will be performed using the general linear model (GLM) after convolving the event onsets with a canonical hemodynamic response function [108]. Both time points will be modeled as two separate sessions within the design matrices. For the EP task, at the subject level we will calculate the interaction between words (self-critical versus standard negative words; the non-words and the negative words will be excluded from the analyses, but used as control variables to ensure cognitive appropriateness) and time (time point 1 versus time point 2) using symbols as baseline. For the SCP task, the subject-level differential *t* contrast will test the interaction between ToM, PUN and time point (the control stimuli will be excluded from the data analysis, but will serve as control for cognitive appropriateness). For both tasks, we will use the one-sample *t* test with the outcomes and arousal changes associated with treatment as regressors for the group-level analyses. The differential contrasts at the group level will test for positive and negative correlation between the interaction at the subject-specific level and BOLD signal changes. Where appropriate, we will control for the corresponding behavioral data from the same assessment point.

### Interim analyses {21b}

No formal stopping rule of the trial is necessary, as the time limit of the recruitment phase (due to funding) will determine when the recruitment is stopped. In addition, we do not anticipate any specific problems that are detrimental to the participants.

### Methods for additional analyses (e.g., sub-group analyses) {20b}

No additional sub-group analyses will be performed.

#### Methods in analysis to handle protocol non-adherence and any statistical methods to handle missing data {20c}

Treatments with low adherence scores will still be included in the trial, but the level of adherence on the named scale will be included as a controlled variable on the level of the statistical analyses. For missing values, we will use classical methods of multiple imputations.

### Plans to give access to the full protocol, participant-level data and statistical code {31c}

Plans to give access to the protocol, participant level-data and statistical code are described in the data management plan.

## Oversight and monitoring

### Composition of the coordinating centre and trial steering committee {5d}

The coordinating center for this RCT is directed by the primary investigator and all co-authors meet at least once a month to oversee advancement of the project; this is also the case for the Trial Steering Committee (TSC). There are three sub-groups within this TSC: (1) clinical sub-group (i.e., therapists and supervisors), (2) research sub-group (i.e., clinical researchers) and (3) fMRI specialists and researchers (i.e., fMRI researchers). There is no specific group related to tasks of the Stakeholder and Public Involvement Group (SPIG). The Ethics Committee does not meet with regard to this trial, except for specific audits or upon request by the Sponsor or the primary investigator{23}.

### Composition of the data monitoring committee, its role and reporting structure {21a}

Given the structure explained under {5d} and the transparent handling of the assessments, it is not necessary to have an additional data monitoring committee.

### Adverse event reporting and harms {22}

The same data management plan outlines procedures to follow in the case of adverse events in the context of the trial, which includes provision, of post-trial care if needed in the case of harm {30}. In particular, no serious adverse events (SAE) are anticipated as a result from the trial or the intervention. Should there be any, they will be reported immediately as required, in terms of expectedness, seriousness, severity and causality.

### Frequency and plans for auditing trial conduct {23}

The Ethics Committee does not meet with regard to this trial, except for specific audits or upon request by the Sponsor or the primary investigator.

### Plans for communicating important protocol amendments to relevant parties (e.g., trial participants, Ethics Committees) {25}

The trial was approved by the competent Ethics Committee (see above) and potential amendments will have to be approved by the same, and be communicated to publishing journals.

### Dissemination plans {31a}

Publications of the results to all relevant groups will be encouraged (i.e., scientific publication, communication at conferences, communication with stakeholders, patients and families).

#### Feasibility: results from the pilot study

We demonstrated the feasibility of the pre-post design [109]. We demonstrated in medication-free, right-handed female patients with BPD (*N* = 8), who were undergoing a 10-session psychiatric treatment, that hypotheses 1 and 2 may be confirmed (due to the small sample size, the analyses for hypotheses 3 and 4 were not tested). The behavioral pre-post treatment outcome effect sizes ranged between *d* = 0.41 (for Outcome Questionaire - 45 (OQ-45)) and *d* = 0.51 (for Borderline Symptom List - 23 (BSL-23)). We observed an increase in arousal within the session of the two-chair dialogue (*d* = 0.36), paralleled by a large decrease in peak arousal between pre-treatment and post-treatment (*d* = 0.80). In the EP task, we demonstrated treatment-associated trends for reduction in neural activity in the associative parts of the putamen when exposed to the individual’s own self-critical words. The exposure to ToM stimuli revealed trends for treatment-related modulation of neural activity in the OFC, ACC and accumbens nucleus (NAcc), and the medio-dorsal nucleus of the thalamus. Neural activity (i.e., in the precuneus, left amygdala) was related to the behavioral changes in arousal, but remained independent from outcome, whereas change in arousal was related to symptom reduction. The feasibility of the trial and relevance of the pre-post hypotheses are therefore demonstrated, and therefore this represents strong justification for the conduct of the proposed RCT. In addition, the effects identified were the basis for the computation of the effect sizes for the trial {14}.

## Discussion

Borderline personality disorder is among the most debilitating and lethal mental disorders. Each year, millions are spent on direct and indirect costs related to this disorder and thousands of people with BPD complete suicide, profoundly impacting the lives of those left behind. There are effective treatments for BPD, but they remain difficult to disseminate. Brief psychiatric treatment is cost-effective and may produce, to some extent, similar effects to a structured psychotherapy program, or at least may represent a promising initial treatment in a stepped-care approach: we think that its implementation should therefore be a major priority in the healthcare system. This is the first study testing the effectiveness of such brief guideline-based psychiatric treatment (compared to treatment as usual) and its underlying mechanisms of change. The latter will be addressed by taking into account the individual’s subjective perspective in the assessment. The integrated methodology optimally compensates for the respective limitations of psychotherapy process and neurofunctional assessments, making it the most scientifically precise, and clinically and ecologically the most relevant approach, to the measurement of mechanisms of change in treatment research. Therefore, this study does not only contribute to the understanding of the effects of treatment as a whole, but also should help render even more effective treatments for BPD by providing a clinically relevant understanding of how change is produced through psychological treatment.

### Trial status

This study is currently ongoing and is not completed (protocol version number 2 from 9 February 2018; start of recruitment 1 December 2018; projected end of recruitment 31 August 2021).

## Data Availability

All co-authors of this of this protocol will have full access to the data. Restrictions to this principle are described in an internal document. The datasets analyzed during the current study are available from the corresponding author on reasonable request and pending ethical approval.

## Abbreviations

ACC: Anterior cingulate cortex
BA: Brodmann area
BOLD: Blood oxygen level dependent
BPD: Borderline personality disorder
BSL-23: Borderline Symptom List – 23
CAMS: Classification of Affective Meaning States
CCRT: Core conflictual relationship theme
DBT: Dialectical behavior therapy
DERS: Difficulties in Emotion Regulation Scale
DSM-5: Diagnostic and statistical manual of mental disorders – 5
EP: Emotional processing
fMRI: Functional magnetic resonance imaging
GLM: General linear model
GPM: Good Psychiatric Management
HLM: Hierarchical linear modelling
IIP: Inventory for Interpersonal Problems
MBT: Mentalization-based therapy
NAcc: Accumbens nucleus
NART: National Adult Reading Test
OFC: Orbito-frontal cortex
OQ-45: Outcome Questionnaire – 45
PD: Personality disorder
PFC: Pre-frontal cortex
PUN: Puns
RCT: Randomized controlled trial
SAM: Self-assessment manikin
SCID-5-CV: Structured Clinical Interview for DSM-5
SCID-5-PD: Structured Clinical Interview for Personality Disorders (DSM-5)
SCP: Socio-cognitive processing
SPIG: Stakeholder and Public Involvement Group
STAXI: State-Trait Anger Expression Inventory
TAU: Treatment as usual
TFP: Transference-focused psychotherapy
ToM: Theory of mind
TSC: Trial Steering Committee
ZAN-BPD: Zanarini Rating Scale for Borderline Personality Disorder
